# Design and Synthesis of Novel Biginelli Adducts: Anticancer Activity and In Silico Study

**DOI:** 10.1002/cbdv.71810

**Published:** 2026-10-12

**Authors:** Aida Iraji, Sara Moghadam Farid, Mohammadreza Eftekharian, Paniz Naseri, Mohammad Mahdavi, Mina Saeedi, Tahmineh Akbarzadeh

**Affiliations:** ^1^ Traditional Medicine and History of Medicine Department of Persian Medicine School of Medicine Shiraz University of Medical Sciences Shiraz Iran; ^2^ Stem Cells Technology Research Center Shiraz University of Medical Sciences Shiraz Iran; ^3^ Endocrinology and Metabolism Research Center Endocrinology and Metabolism Clinical Sciences Institute Tehran University of Medical Sciences Tehran Iran; ^4^ Student Research Committee Jahrom University of Medical Sciences Jahrom Iran; ^5^ Department of Medicinal Chemistry Faculty of Pharmacy Tehran University of Medical Sciences Tehran Iran; ^6^ Medicinal Plants Research Center Faculty of Pharmacy Tehran University of Medical Sciences Tehran Iran; ^7^ Persian Medicine and Pharmacy Research Center Tehran University of Medical Sciences Tehran Iran

**Keywords:** apoptosis, Biginelli adducts, breast cancer, G2/M arrest

## Abstract

In this study, we report the synthesis of 14 Biginelli adducts and the evaluation of their anticancer activity against three human cancer cell lines: MCF‐7 (breast), SW480 (colon), and Hep‐G2 (liver), as well as the noncancerous Hek‐293 cell line. Among the synthesized derivatives, compound **8n** emerged as the most potent, inducing significant cytotoxicity and apoptosis in MCF‐7 cells. Structure–activity relationship (SAR) analysis showed that specific modifications, such as methyl and nitro substitutions, enhanced anticancer activity, whereas bulky substituents reduced potency. Further investigations confirmed that compound **8n** induces cell cycle arrest at the S and G2/M phases, highlighting its antiproliferative mechanism. Molecular docking and molecular dynamics simulations suggest that compound **8n** could bind to Eg5, a potential regulator of cell division. However, this study did not include direct biochemical testing of the protein target (Eg5).

## Graphical abstract

Compound **8n** emerged as the most potent agent against MCF‐7 cells, inducing apoptosis and cell cycle arrest. Molecular docking identified Eg5 kinesin as a potential target, highlighting these derivatives as promising leads for cancer therapeutics.

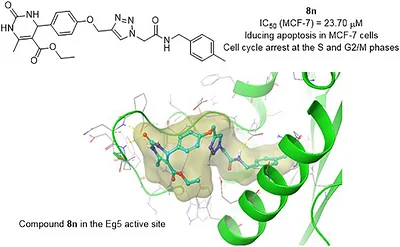

## 1 Introduction

Cancer remains one of the leading causes of death globally, with its prevalence rising steadily. This neoplastic disease is characterized by dysregulated cellular proliferation, invasion, and metastasis, primarily driven by genetic and epigenetic alterations [1, 2]. Chemotherapy, alongside surgery and radiotherapy, constitutes the mainstay of cancer treatment in clinical practice. Conventional chemotherapeutic agents exert their effects by disrupting cell cycle progression, DNA replication, or mitotic spindle formation, ultimately inducing apoptosis in rapidly proliferating cells [3, 4].

The emergence of drug resistance presents a major challenge, rendering chemotherapy less effective in managing this disease. Medicinal chemistry researchers are actively developing new anticancer agents that target specific cancer cell subtypes. Breast cancer remains a critical health concern for women worldwide, with millions diagnosed annually. The complex biological mechanisms underlying breast cancer, combined with factors such as genetic mutations, hormonal influences, and environmental exposures, contribute to its clinical complexity and high mortality rate [5].

Taxanes and vinca alkaloids, as microtubule‐targeting agents, have proven effective at inhibiting cancer cell proliferation. Taxanes, including paclitaxel, docetaxel, and cabazitaxel (Figure 1), stabilize microtubules, preventing their depolymerization [6, 7]. This mechanism arrests cell division and ultimately induces apoptosis. Similarly, vinca alkaloids such as vincristine, vinblastine, vindesine, and vinorelbine (Figure 1) bind to tubulin, inhibiting microtubule polymerization. This action also arrests mitosis, leading to cell death [8].

**FIGURE 1:**
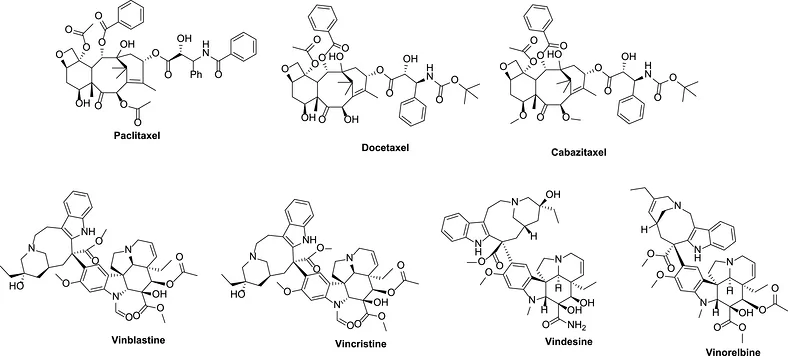
The structure of taxanes and vinca alkaloids.

A promising therapeutic strategy involves targeting kinesin spindle protein (KSP), also known as Eg5. Eg5 plays a pivotal role in spindle assembly and chromosome segregation during mitosis, making it a prime target for anticancer research [9]. Given that cancer cells proliferate more rapidly than normal cells, Eg5 inhibition causes selective mitotic catastrophe, targeting aggressive malignancies such as breast and colon cancer [10, 11]. Studies indicate that Eg5 inhibitors trigger apoptotic pathways [12] and induce cell cycle arrest at the G2/M phase, halting proliferation [13, 14]. In this work, molecular docking and molecular dynamics simulations revealed that compound **8n** may bind to the Eg5 kinesin protein, a probable regulator of cell division. However, this study did not include a direct biochemical test to confirm compound **8n** binding to the protein.

The Biginelli reaction, a multicomponent condensation involving an aldehyde, a β‐ketoester and urea or thiourea, efficiently yields dihydropyrimidinones (DHPMs). This reaction has attracted considerable interest due to its synthetic efficiency and the generation of bioactive scaffolds exhibiting antiviral, antibacterial, anti‐inflammatory, and anticancer properties [15].

Notable examples include Monastrol (**A**, Figure 2), which shows effective anticancer potency and selectivity against the kinesin Eg5 motor protein, essential for spindle bipolarity [16]. Piperastrol (**B**, Figure 2) demonstrated potent anticancer activity by inhibiting the growth of MCF‐7 breast, 786‐O kidney, and OVCAR03 ovarian cancer cells at low concentrations, highlighting the therapeutic promise of DHPM‐based compounds [17, 18]. Toolabi et al. synthesized various 3,4‐dihydropyrimidine derivatives via the Biginelli reaction and evaluated their cytotoxicity against MCF‐7, A549, and HeLa cancer cells. Among the synthesized analogs, compound **C** (Figure 2), bearing a 4‐chloro‐aryl group at the C4 position, showed the strongest activity, particularly against A549 cells (IC_50_ = 18.65 µM), inducing apoptosis and demonstrating in silico interactions with the Eg5 protein [19]. Additionally, compound **D** (Figure 2), another chlorine‐substituted tetrahydropyrimidine, displayed exceptional potency in K562 cells (IC_50_ = 1.66 µM) and modulated gene and miRNA expression profiles, underscoring its potential as a promising anticancer candidate [20].

**FIGURE 2:**
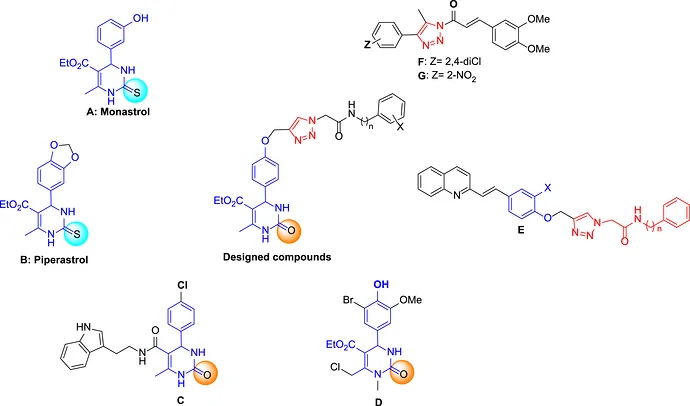
Design strategy of target compounds based on Biginelli adducts and 1,2,3‐triazole pharmacophores.

In addition, the 1,2,3‐triazole scaffold, typically obtained through the copper‐catalyzed azide–alkyne cycloaddition (CuAAC, or “Click reaction”), has emerged as a privileged pharmacophore in medicinal chemistry. This reaction provides high regioselectivity, good yields, and mild reaction conditions, making it an efficient tool for molecular hybridization and late‐stage functionalization of bioactive molecules [21, 22]. Triazole hybrids exhibit multitargeted mechanisms in cancer progression and overcome multidrug resistance with minimized side effects [23]. In recent work, a series of quinoline‐vinyl‐phenoxy‐1,2,3‐triazole‐acetamide hybrids were synthesized targeting breast cancer; compound **E** showed the strongest cytotoxicity against MCF‐7 and MDA‐MB‐231 cells (IC_50_ = 16.84 and 21.78 µM) and induced apoptosis and caspase‐3 activation, identifying apoptosis as its primary anticancer mechanism [24]. Salama et al. designed and synthesized a series of 1,2,3‐triazole hybrids as promising anticancer agents targeting the EGFR/mTOR signaling pathway and tubulin polymerization. Evaluation against nine panels of human cancer cell lines comprising a total of 60 lines exhibited strong cytotoxicity; compounds **F** and **G** were the most potent analogs, showing more than 80% growth inhibition, especially in melanoma and colorectal cancer, while maintaining minimal toxicity toward normal colon cells. Both compounds displayed nanomolar GI_50_ values (0.00169–0.629 nM), induced G1/S phase cell cycle arrest, and improved apoptosis by elevating Bax to Bcl‐2 as well as caspase 3 and 9 activity [25].

This study employed a hybridization strategy to design and synthesize a new series of tetrahydropyrimidinones linked to 1,2,3‐triazoles. Initially, all analogs were evaluated against MCF‐7, SW480, and Hep‐G2 cell lines. We further assessed these derivatives using cell cycle analysis, apoptosis assays, and molecular docking and dynamics simulations to elucidate their mechanisms of action.

## 2 Materials and Methods

### 2.1 General

All chemicals and solvents were purchased from Merck and Aldrich and were used without further purification. Melting points were determined using a Kofler hot stage apparatus and are reported uncorrected. Infrared (IR) spectra were recorded on a Nicolet Magna FTIR 550 spectrophotometer as KBr pellets. ^1^H and ^13^C NMR were acquired on an INOVA 500 MHz spectrometer (Varian), using tetramethylsilane (TMS) as the internal standard. Elemental analyses were performed on an Elementar Analysensysteme GmbH VarioEL CHNS analyzer. Mass spectra were recorded on an Agilent Technology (HP) mass spectrometer operating at an ionization potential of 70 eV. Analytical HPLC was performed on a YL9100 HPLC system (Young Lin, Korea) equipped with a UV detector. Separation was achieved using a reversed‐phase column (Teknokroma, C18, 5 µm, 150 × 4.6 mm) and the mobile phase consisted of methanol (solvent A) and water, a gradient of 0%–100% solvent A in 9 min, 1 min at 40%, to 100% within 1 min, to 100% at 5 min, to 40% within 0.1 min (total run time 9 min); flow rate, 1 mL/min; detection, 254 nm; injection volume, 20 µL.

### 2.2 Synthesis

#### 2.2.1 General Procedure for the Synthesis of Compound **4**

A mixture of urea (**1**, 1 mmol), 4‐(prop‐2‐yn‐1‐yloxy)benzaldehyde (**2**, 1 mmol), ethyl acetoacetate (**3**, 1 mmol), and a catalytic amount of hydrochloric acid was refluxed in acetic acid (15 mL) for 5 h. Upon completion of the reaction, as monitored by TLC, the mixture was allowed to cool to room temperature and then poured into ice water. The resulting precipitate was collected by filtration, washed with water, and dried to afford compound **4**.

#### 2.2.2 General Procedure for the Synthesis of Compound **8a‐n**

A mixture of compound **7a‐n** (1.1 mmol), NaN_3_ (0.9 mmol), and NEt_3_ (1.3 mmol) in DMF (4 mL) was stirred at room temperature for 30 min to generate the corresponding azide intermediate in situ. Subsequently, compound **4** (1 mmol) and catalytic amounts of CuSO_4_·5H_2_O (0.02 g) and sodium ascorbate (SA) were added, and the mixture was stirred for 24 h. TLC monitored the reaction progress. Upon completion, the reaction mixture was poured into ice water. The resulting precipitates were collected by filtration, washed with water, and dried to afford compounds **8a‐n**. The products were obtained in good purity; however, recrystallization from EtOH was carried out to remove minor impurities when necessary.

##### 2.2.2.1 Ethyl‐6‐methyl‐2‐oxo‐4‐(4‐((1‐(2‐oxo‐2‐(phenylamino)ethyl)‐1*H*‐1,2,3‐triazol‐4‐yl)methoxy)phenyl)‐1,2,3,4‐tetrahydropyrimidine‐5‐carboxylate (**8a**)

Cream‐colored powder, Yield: 74%, mp 128°C–132°C. IR (KBr): 3245 (NH), 3115 (CH), 2925 (CH), 2850 (CH), 1726 (C═O), 1694 (C═O), 1661 (C═C) cm^−1^. ^1^HNMR (500 MHz, DMSO‐*d_6_*): 10.49 (s, 1H, NH), 9.16 (s, 1H, NH), 8.26 (s, 1H, NH), 7.69 (s, 1H, triazole), 7.59 (d, *J* = 7.8 Hz, 2H, H2, H6), 7.35 (t, *J* = 7.8 Hz, 2H, H3, H5), 7.18 (d, *J* = 8.7 Hz, 2H, H3′, H5′), 7.10 (t, *J* = 7.8 Hz, 1H, H4), 7.02 (d, *J* = 8.7 Hz, 2H, H2′, H6′), 5.36 (s, 2H, CH_2_), 5.16 (s, 2H, CH_2_), 5.12 (d, *J* = 3.3 Hz, 1H, CH), 3.99 (q, *J* = 7.1 Hz, 2H, CH_2_), 2.26 (s, 3H, CH_3_), 1.12 (t, *J* = 7.1 Hz, 3H, CH_3_) ppm. ^13^CNMR (125 MHz, DMSO‐*d_6_*): 165.8, 164.6, 157.7, 152.6, 148.5, 142.9, 138.9, 137.9, 129.4, 127.9, 126.7, 124.3, 119.7, 114.9, 100.0, 61.5, 59.6, 53.8, 52.7, 18.3, 14.6 ppm. Calcd for C_25_H_26_N_6_O_5_: C, 61.22; H, 5.34; N, 17.13. Found: C, 61.24; H, 5.21; N, 17.37.

##### 2.2.2.2 Ethyl‐6‐methyl‐2‐oxo‐4‐(4‐((1‐(2‐oxo‐2‐(*p*‐tolylamino)ethyl)‐1*H*‐1,2,3‐triazol‐4‐yl)methoxy)phenyl)‐1,2,3,4‐tetrahydropyrimidine‐5‐carboxylate (**8b**)

Cream‐colored powder, Yield: 77%, mp 121°C–123°C. IR (KBr): 3245 (NH), 3115 (CH), 2925 (CH), 2850 (CH), 1726 (CO), 1695 (C═O), 1660 (C═C) cm^−1^. ^1^HNMR (500 MHz, DMSO‐*d_6_*): 10.40 (s, 1H, NH), 9.16 (s, 1H, NH), 8.25 (s, 1H, NH), 7.69 (s, 1H, triazole), 7.48 (d, *J* = 8.5 Hz, 2H, H2, H6), 7.18‐7.14 (m, 5H, H3, H4, H5, H3′, H5′), 7.01 (d, *J* = 8.9 Hz, 2H, H2′, H6′), 5.33 (s, 2H, CH_2_), 5.15 (s, 2H, CH_2_), 5.11 (d, *J* = 3.3 Hz, 1H, CH), 4.00 (q, *J* = 7.0 Hz, 2H, CH_2_), 2.27‐2.26 (m, 6H, 2×CH_3_), 1.12 (t, *J* = 7.0 Hz, 3H, CH_3_) ppm. ^13^CNMR (125 MHz, DMSO‐*d_6_*): 165.8, 164.4, 157.7, 152.6, 148.5, 14.9., 137.9, 136.4, 133.2, 129.7, 127.9, 126.7, 119.7, 114.9, 100.0, 61.5, 59.6, 53.8, 52.6, 20.9, 18.2, 14.6 ppm. Calcd for C_26_H_28_N_6_O_5_: C, 61.89; H, 5.59; N, 16.66. Found: C, 61.94; H, 5.41; N, 16.61.

##### 2.2.2.3 Ethyl‐4‐(4‐((1‐(2‐((2,3‐dimethylphenyl)amino)‐2‐oxoethyl)‐1*H*‐1,2,3‐triazol‐4‐yl)methoxy)phenyl)‐6‐methyl‐2‐oxo‐1,2,3,4‐tetrahydropyrimidine‐5‐carboxylate (**8c**)

Cream‐colored powder, Yield: 69%, mp 131°C–135°C. IR (KBr): 3245 (NH), 3115 (CH), 2925 (CH), 2850 (CH), 1724 (C═O), 1694 (C═O), 1660 (C═C) cm^−1^. ^1^HNMR (500 MHz, DMSO‐*d_6_*): 9.88 (s, 1H, NH), 9.17 (s, 1H, NH), 8.26 (s, 1H, NH), 7.70 (s, 1H, triazole), 7.20–7.18 (m, 3H, H2, H3′, H5′), 7.09–7.01 (m, 4H, H3, H4, H2′, H6′), 5.40 (s, 2H, CH_2_), 5.16 (s, 2H, CH_2_), 5.13 (d, *J* = 3.4 Hz, 1H, CH), 4.00 (q, *J* = 7.1 Hz, 2H, CH_2_), 2.68 (s, 6H, 2×CH_3_), 2.12 (s, 3H, CH_3_), 1.12 (t, *J* = 7.1 Hz, 3H, CH_3_) ppm. ^13^CNMR (125 MHz, DMSO‐*d_6_*): 165.9, 164.9, 157.7, 152.7, 148.5, 143.0, 137.9, 137.6, 135.7, 131.5, 127.9, 127.8, 126.7, 125.8, 123.7, 114.9, 100.0, 61.5, 59.6, 53.8, 52.4, 20.6, 18.3, 14.6, 14.5 ppm. MS (*m/z*, %): 518 (M^+^, 2), 161 (9), 105 (16), 80 (100). Calcd for C_27_H_30_N_6_O_5_: C, 62.54; H, 5.83; N, 16.21. Found: C, 62.69; H, 5.75; N, 16.38.

##### 2.2.2.4 Ethyl‐4‐(4‐((1‐(2‐((2,6‐dimethylphenyl)amino)‐2‐oxoethyl)‐1*H*‐1,2,3‐triazol‐4‐yl)methoxy)phenyl)‐6‐methyl‐2‐oxo‐1,2,3,4‐tetrahydropyrimidine‐5‐carboxylate (**8d**)

Cream‐colored powder, Yield: 78%, mp 115°C–119°C. IR (KBr): 3246 (NH), 3112 (CH), 2925 (CH), 2850 (CH), 1726 (C═O), 1694 (C═O), 1660 (C═C) cm^−1^. ^1^HNMR (500 MHz, DMSO‐*d_6_*): 9.79 (s, 1H, NH), 9.18 (s, 1H, NH), 8.26 (s, 1H, NH), 7.70 (s, 1H, triazole), 7.18 (d, *J* = 6.7 Hz, 2H, H3′, H5′), 7.10–7.09 (m, 3H, H3, H4, H5), 7.01 (d, *J* = 6.7 Hz, 2H, H2′, H6′), 5.41 (s, 2H, CH_2_), 5.16 (s, 2H, CH_2_), 5.13 (s, 1H, CH), 4.00 (q, *J* = 7.1 Hz, 2H, CH_2_), 2.27 (s, 3H, CH_3_), 2.17 (s, 6H, 2×CH_3_), 1.12 (t, *J* = 7.1 Hz, 3H, CH_3_) ppm. ^13^CNMR (125 MHz, DMSO‐*d_6_*): 165.8, 164.5, 157.7, 152.6, 148.5, 143.0, 137.9, 135.6, 134.7, 128.2, 127.9, 127.2, 126.6, 114.9, 100.0, 61.5, 59.6, 53.8, 52.1, 18.5, 18.3, 14.6 ppm. MS (*m/z*, %): 518 (M^+^, 2), 149 (6), 80 (100). Calcd for C_27_H_30_N_6_O_5_: C, 62.54; H, 5.83; N, 16.21. Found: C, 62.58; H, 5.71; N, 16.19.

##### 2.2.2.5 Ethyl‐4‐(4‐((1‐(2‐((4‐ethylphenyl)amino)‐2‐oxoethyl)‐1*H*‐1,2,3‐triazol‐4‐yl)methoxy)phenyl)‐6‐methyl‐2‐oxo‐1,2,3,4‐tetrahydropyrimidine‐5‐carboxylate (**8e**)

Cream‐colored powder, Yield: 79%, mp 119°C–122°C. IR (KBr): 3245 (NH), 3115 (CH), 2925 (CH), 2851 (CH), 1726 (C═O), 1696 (C═O), 1662 (C═C) cm^−1^. ^1^HNMR (500 MHz, DMSO‐*d_6_*): 10.41 (s, 1H, NH), 9.17 (s, 1H, NH), 8.26 (s, 1H, NH), 7.69 (s, 1H, triazole), 7.50 (d, *J* = 8.1 Hz, 2H, H2, H6), 7.19–7.17 (m, 4H, H3, H5, H3′, H5′), 7.01 (d, *J* = 8.8 Hz, 2H, H2′, H6′), 5.34 (s, 2H, CH_2_), 5.16 (s, 2H, CH_2_), 5.12 (s, 1H, CH), 4.00 (q, *J* = 7.1 Hz, 2H, CH_2_), 2.57 (q, *J* = 7.5 Hz, 2H, CH_2_), 2.26 (s, 3H, CH_3_), 1.17 (t, *J* = 7.5 Hz, 3H, CH_3_), 1.12 (t, *J* = 7.1 Hz, 3H, CH_3_) ppm. ^13^CNMR (125 MHz, DMSO‐*d_6_*): 165.8, 164.4, 157.7, 152.6, 148.5, 143.0, 139.7, 137.9, 136.3, 128.6, 127.9, 126.7, 119.8, 114.9, 100.0, 61.5, 59.6, 53.8, 52.7, 28.1, 18.2, 16.1, 14.6 ppm. Calcd for C_27_H_30_N_6_O_5_: C, 62.54; H, 5.83; N, 16.21. Found: C, 62.37; H, 5.51; N, 16.18.

##### 2.2.2.6 Ethyl‐4‐(4‐((1‐(2‐((4‐methoxyphenyl)amino)‐2‐oxoethyl)‐1*H*‐1,2,3‐triazol‐4‐yl)methoxy)phenyl)‐6‐methyl‐2‐oxo‐1,2,3,4‐tetrahydropyrimidine‐5‐carboxylate (**8f**)

Cream‐colored powder, Yield: 82%, mp 116°C–119°C. IR (KBr): 3245 (NH), 3115 (CH), 2925 (CH), 2850 (CH), 1728 (C═O), 1694 (C═O), 1662 (C═C) cm^−1^. ^1^HNMR (500 MHz, DMSO‐*d_6_*): 10.34 (s, 1H, NH), 9.16 (s, 1H, NH), 8.25 (s, 1H, NH), 7.68 (s, 1H, triazole), 7.50 (d, *J* = 8.5 Hz, 2H, H2, H6), 7.17 (d, *J* = 8.0 Hz, 2H, H3′, H5′), 7.01 (d, *J* = 8.0 Hz, 2H, H2′, H6′), 6.92 (d, *J* = 8.5 Hz, 2H, H3, H5), 5.32 (s, 2H, CH_2_), 5.15 (s, 2H, CH_2_), 5.11 (d, *J* = 3.4 Hz, 1H, CH), 4.00 (q, *J* = 7.1 Hz, 2H, CH_2_), 3.74 (s, 3H, OCH_3_), 2.26 (s, 3H, CH_3_), 1.12 (t, *J* = 7.1 Hz, 3H, CH_3_) ppm. ^13^CNMR (125 MHz, DMSO‐*d_6_*): 165.8, 164.1, 157.7, 156.0, 152.6, 148.5, 142.9, 137.9, 132.0, 127.9, 126.7, 121.2, 114.9, 114.5, 100.0, 61.5, 59.6, 55.6, 53.8, 52.6, 18.2, 14.6 ppm. Calcd for C_26_H_28_N_6_O_6_: C, 59.99; H, 5.42; N, 16.14. Found: C, 60.17; H, 5.21; N, 16.35.

##### 2.2.2.7 Ethyl‐6‐methyl‐4‐(4‐((1‐(2‐((4‐nitrophenyl)amino)‐2‐oxoethyl)‐1*H*‐1,2,3‐triazol‐4‐yl)methoxy)phenyl)‐2‐oxo‐1,2,3,4‐tetrahydropyrimidine‐5‐carboxylate (**8g**)

Yellow powder, Yield: 72%, mp 125°C–127°C. IR (KBr): 3245 (NH), 3115 (CH), 2925 (CH), 2852 (CH), 1725 (C═O), 1694 (C═O), 1662 (C═C), 1575 (NO_2_), 1375 (NO_2_) cm^−1^. ^1^HNMR (500 MHz, DMSO‐*d_6_*): 11.10 (s, 1H, NH), 9.17 (s, 1H, NH), 8.29–8.25 (m, 3H, NH, H3, H5), 7.84 (d, *J* = 8.8 Hz, 2H, H2, H6), 7.69 (s, 1H, triazole), 7.18 (d, *J* = 8.5 Hz, 2H, H3′, H5′), 7.02 (d, *J* = 8.5 Hz, 2H, H2′, H6′), 5.45 (s, 2H, CH_2_), 5.17 (s, 2H, CH_2_), 5.12 (s, 1H, CH), 4.00 (q, *J* = 7.1 Hz, 2H, CH_2_), 2.26 (s, 3H, CH_3_), 1.12 (t, *J* = 7.1 Hz, 3H, CH_3_) ppm. ^13^CNMR (125 MHz, DMSO‐*d_6_*): 165.9, 165.8, 157.7, 152.6, 148.5, 146.7, 145.0, 143.1, 137.9, 127.9, 126.7, 125.6, 119.5, 114.9, 100.0, 61.5, 59.6, 53.8, 52.8, 18.2, 14.6 ppm. MS (*m/z*, %): 535 (M^+^, 2), 279 (11), 149 (100), 55 (58). Calcd for C_25_H_25_N_7_O_7_: C, 56.07; H, 4.71; N, 18.31. Found: C, 56.13; H, 4.58; N, 18.10.

##### 2.2.2.8 Ethyl‐6‐methyl‐4‐(4‐((1‐(2‐((2‐methyl‐4‐nitrophenyl)amino)‐2‐oxoethyl)‐1*H*‐1,2,3‐triazol‐4‐yl)methoxy)phenyl)‐2‐oxo‐1,2,3,4‐tetrahydropyrimidine‐5‐Carboxylate (**8h**)

Yellow powder, Yield: 67%, mp 120°C–124°C. IR (KBr): 3248 (NH), 3115 (CH), 2925 (CH), 2850 (CH), 1725 (C═O), 1694 (C═O), 1662 (C═C), 1575 (NO_2_), 1375 (NO_2_) cm^−1^. ^1^HNMR (500 MHz, DMSO‐*d_6_*): 10.08 (s, 1H, NH), 9.16 (s, 1H, NH), 8.28 (s, 1H, NH), 8.18 (s, 1H, H3), 8.09 (d, *J* = 8.8 Hz, 1H, H5), 7.96 (d, *J* = 8.8 Hz, 1H, H6), 7.69 (s, 1H, triazole), 7.17 (d, *J* = 8.5 Hz, 2H, H3′, H5′), 7.01 (d, *J* = 8.5 Hz, 2H, H2′, H6′), 5.52 (s, 2H, CH_2_), 5.17 (s, 2H, CH_2_), 5.11 (s, 1H, CH), 3.99 (q, *J* = 7.0 Hz, 2H, CH_2_), 2.42 (s, 3H, CH_3_), 2.26 (s, 3H, CH_3_), 1.12 (t, *J* = 7.0 Hz, 3H, CH_3_) ppm. ^13^CNMR (125 MHz, DMSO‐*d_6_*): 165.8, 165.7, 157.7, 152.6, 148.5, 144.0, 143.1, 142.6, 137.9, 131.8, 127.9, 126.7, 126.0, 123.7, 122.3, 114.9, 100.0, 61.5, 59.6, 53.8, 52.6, 18.3, 18.2, 14.6 ppm. MS (*m/z*, %): 549 (M^+^, 2), 276 (22), 247 (100), 203 (74), 155 (44), 64 (99). Calcd for C_26_H_27_N_7_O_7_: C, 56.83; H, 4.95; N, 17.84. Found: C, 56.71; H, 4.75; N, 17.93.

##### 2.2.2.9 Ethyl‐4‐(4‐((1‐(2‐((2‐fluorophenyl)amino)‐2‐oxoethyl)‐1*H*‐1,2,3‐triazol‐4‐yl)methoxy)phenyl)‐6‐methyl‐2‐oxo‐1,2,3,4‐tetrahydropyrimidine‐5‐carboxylate (**8i**)

Cream‐colored powder, Yield: 80%, mp 114°C–116°C. IR (KBr): 3245 (NH), 3119 (CH), 2925 (CH), 2852 CH), 1725 (C═O), 1698 (C═O), 1662 (C═C) cm^−1^. ^1^HNMR (500 MHz, DMSO‐*d_6_*): 10.33 (s, 1H, NH), 9.16 (s, 1H, NH), 8.26 (s, 1H, NH), 7.94–7.90 (m, 1H, H6), 7.69 (s, 1H, triazole), 7.33–7.28 (m, 1H, H3), 7.20–7.17 (m, 4H, H4, H5, H3′, H5′), 7.01 (d, *J* = 8.7 Hz, 2H, H2′, H6′), 5.45 (s, 2H, CH_2_), 5.16 (s, 2H, CH_2_), 5.12 (d, *J* = 3.0 Hz, 1H, CH), 4.00 (q, *J* = 7.0 Hz, 2H, CH_2_), 2.26 (s, 3H, CH_3_), 1.12 (t, *J* = 7.0 Hz, 3H, CH_3_) ppm. ^13^CNMR (125 MHz, DMSO‐*d_6_*): 165.9, 165.3, 157.7, 153.9 (d, *J_C‐F_* = 243.9 Hz), 152.6, 148.5, 143.0, 137.9, 127.9, 126.7, 126.1 (d, *J_C‐F_* = 7.4 Hz), 125.9 (d, *J_C‐F_* = 12.1 Hz), 125.0 (d, *J_C‐F_* = 3.35 Hz), 124.2, 116.1 (d, *J_C‐F_* = 19.1 Hz), 114.9, 100.0, 61.5, 59.6, 53.8, 52.5, 18.2, 14.6 ppm. MS (*m/z*, %): 508 (M^+^, 2), 149 (17), 80 (100). Calcd for C_25_H_25_FN_6_O_5_: C, 59.05; H, 4.96; N, 16.53. Found: C, 59.25; H, 4.87; N, 16.21.

##### 2.2.2.10 Ethyl‐4‐(4‐((1‐(2‐((4‐chlorophenyl)amino)‐2‐oxoethyl)‐1*H*‐1,2,3‐triazol‐4‐yl)methoxy)phenyl)‐6‐methyl‐2‐oxo‐1,2,3,4‐tetrahydropyrimidine‐5‐carboxylate (**8j**)

Cream‐colored powder, Yield: 71%, mp 111°C–115°C. IR (KBr): 3245 (NH), 3117 (CH), 2925 (CH), 2852 (CH), 1725 (C═O), 1694 (C═O), 1665 (C═C) cm^−1^. ^1^HNMR (500 MHz, DMSO‐*d_6_*): 10.63 (s, 1H, NH), 9.16 (s, 1H, NH), 8.26 (s, 1H, NH), 7.69 (s, 1H, triazole), 7.62 (d, *J* = 8.8 Hz, 2H, H2, H6), 7.40 (t, *J* = 8.8 Hz, 2H, H3, H5), 7.17 (d, *J* = 8.7 Hz, 2H, H3′, H5′), 7.01 (d, *J* = 8.7 Hz, 2H, H2′, H6′), 5.36 (s, 2H, CH_2_), 5.16 (s, 2H, CH_2_), 5.11 (s, 1H, CH), 4.00 (q, *J* = 7.1 Hz, 2H, CH_2_), 2.26 (s, 3H, CH_3_), 1.12 (t, *J* = 7.1 Hz, 3H, CH_3_) ppm. ^13^CNMR (125 MHz, DMSO‐*d_6_*): 165.8, 164.9, 157.7, 152.6, 148.5, 143.0, 137.9, 137.8, 129.3, 127.9, 127.8, 126.7, 121.3, 114.9, 100.0, 61.5, 59.6, 53.8, 52.7, 18.2, 14.6 ppm. Calcd for C_25_H_25_ClN_6_O_5_: C, 57.20; H, 4.80; N, 16.01. Found: C, 57.34; H, 4.97; N, 16.18.

##### 2.2.2.11 Ethyl‐4‐(4‐((1‐(2‐((2,4‐dichlorophenyl)amino)‐2‐oxoethyl)‐1*H*‐1,2,3‐triazol‐4‐yl)methoxy)phenyl)‐6‐methyl‐2‐oxo‐1,2,3,4‐tetrahydropyrimidine‐5‐carboxylate (**8k**)

Cream‐colored powder, Yield: 75%, mp 125°C–128°C. IR (KBr): 3245 (NH), 3117 (CH), 2925 (CH), 2850 (CH), 1725 (C═O), 1695 (C═O), 1665 (C═C) cm^−1^. ^1^HNMR (500 MHz, DMSO‐*d_6_*): 10.17 (s, 1H, NH), 9.16 (s, 1H, NH), 8.26 (s, 1H, NH), 7.80 (d, *J* = 8.8 Hz, 1H, H6), 7.71 (d, *J* = 2.5 Hz, 1H, H3), 7.69 (s, 1H, triazole), 7.44 (dd, *J* = 2.5, 8.8 Hz, 1H, H5), 7.17 (d, *J* = 8.8 Hz, 2H, H3′, H5′), 7.01 (d, *J* = 8.8 Hz, 2H, H2′, H6′), 5.48 (s, 2H, CH_2_), 5.16 (s, 2H, CH_2_), 5.11 (d, *J* = 3.4 Hz, 1H, CH), 3.99 (q, *J* = 7.1 Hz, 2H, CH_2_), 2.26 (s, 3H, CH_3_), 1.12 (t, *J* = 7.1 Hz, 3H, CH_3_) ppm. ^13^CNMR (125 MHz, DMSO‐*d_6_*): 165.8, 165.5, 157.7, 152.6, 148.5, 143.0, 137.9, 133.9, 130.3, 129.6, 128.2, 127.9, 127.6, 127.3, 126.7, 114.9, 100.0, 61.4, 59.6, 53.8, 52.4, 18.2, 14.6 ppm. Calcd for C_25_H_24_Cl_2_N_6_O_5_: C, 53.68; H, 4.32; N, 15.02. Found: C, 53.46; H, 4.28; N, 15.27.

##### 2.2.2.12 Ethyl‐4‐(4‐((1‐(2‐((4‐bromophenyl)amino)‐2‐oxoethyl)‐1*H*‐1,2,3‐triazol‐4‐yl)methoxy)phenyl)‐6‐methyl‐2‐oxo‐1,2,3,4‐tetrahydropyrimidine‐5‐carboxylate (**8l**)

Cream‐colored powder, Yield: 65%, mp 112°C–114°C. IR (KBr): 3245 (NH), 3115 (CH), 2925 (CH), 2852 (CH), 1725 (C═O), 1694 (C═O), 1666 (C═C) cm^−1^. ^1^HNMR (500 MHz, DMSO‐*d_6_*): 10.63 (s, 1H, NH), 9.17 (s, 1H, NH), 8.26 (s, 1H, NH), 7.69 (s, 1H, triazole), 7.58 (d, *J* = 8.9 Hz, 2H, H2, H6), 7.53 (d, *J* = 8.9 Hz, 2H, H3, H5), 7.18 (d, *J* = 8.6 Hz, 2H, H3′, H5′), 7.02 (d, *J* = 8.6 Hz, 2H, H2′, H6′), 5.37 (s, 2H, CH_2_), 5.16 (s, 2H, CH_2_), 5.12 (d, *J* = 3.6 Hz, 1H, CH), 4.00 (q, *J* = 7.1 Hz, 2H, CH_2_), 2.26 (s, 3H, CH_3_), 1.12 (t, *J* = 7.1 Hz, 3H, CH_3_) ppm. ^13^CNMR (125 MHz, DMSO‐*d_6_*): 165.8, 164.9, 157.7, 152.6, 148.5, 143.0, 138.3, 137.9, 132.2, 127.9, 126.7, 121.6, 115.9, 114.9, 100.0, 61.5, 59.6, 53.8, 52.7, 18.3, 14.6 ppm. Calcd for C_25_H_25_BrN_6_O_5_: C, 52.73; H, 4.43; N, 14.76. Found: C, 52.81; H, 4.24; N, 14.92.

##### 2.2.2.13 Ethyl‐4‐(4‐((1‐(2‐(benzylamino)‐2‐oxoethyl)‐1*H*‐1,2,3‐triazol‐4‐yl)methoxy)phenyl)‐6‐methyl‐2‐oxo‐1,2,3,4‐tetrahydropyrimidine‐5‐carboxylate (**8m**)

Cream‐colored powder, Yield: 84%, mp 126°C–130°C. IR (KBr): 3245 (NH), 3115 (CH), 2925 (CH), 2852 (CH), 1725 (C═O), 1695 (C═O), 1660 (C═O) cm^−1^. ^1^HNMR (500 MHz, DMSO‐*d_6_*): 9.17 (s, 1H, NH), 8.85 (t, *J* = 6.0 Hz, 1H, NH), 8.21 (s, 1H, NH), 7.69 (s, 1H, triazole), 7.37–7.34 (m, 2H, H3, H5), 7.31–7.26 (m, 3H, H2, H4, H6), 7.18 (d, *J* = 7.7 Hz, 2H, H3′, H5′), 7.1 (d, *J* = 7.7 Hz, 2H, H2′, H6′), 5.20 (s, 2H, CH_2_), 5.14 (s, 2H, CH_2_), 5.12 (s, 1H, CH), 4.35 (d, *J* = 6.0 Hz, 2H, CH_2_), 4.00 (q, *J* = 7.1 Hz, 2H, CH_2_), 2.26 (s, 3H, CH_3_), 1.12 (t, *J* = 7.1 Hz, 3H, CH_3_) ppm. ^13^CNMR (125 MHz, DMSO‐*d_6_*): 165.9, 165.8, 157.7, 152.6, 148.5, 142.9, 139.2, 137.9, 128.8, 128.0, 127.9, 127.5, 126.6, 114.9, 100.0, 61.5, 59.6, 53.8, 52.1, 42.9, 18.3, 14.6 ppm. MS (*m/z*, %): 504 (M^+^, 2), 247 (34), 183 (23), 155 (13), 80 (100). Calcd for C_26_H_28_N_6_O_5_: C, 61.89; H, 5.59; N, 16.66. Found: C, 61.93; H, 5.68; N, 16.81.

##### 2.2.2.14 Ethyl‐6‐methyl‐4‐(4‐((1‐(2‐((4‐methylbenzyl)amino)‐2‐oxoethyl)‐1*H*‐1,2,3‐triazol‐4‐yl)methoxy)phenyl)‐2‐oxo‐1,2,3,4‐tetrahydropyrimidine‐5‐carboxylate (**8n**)

Cream‐colored powder, Yield: 81%, mp 120°C–122°C. IR (KBr): 3245 (NH), 3115 (CH), 2928 (CH), 2850 (CH), 1725 (C═O), 1695 (C═O), 1660 (C═C) cm^−1^. ^1^HNMR (500 MHz, DMSO‐*d_6_*): 9.18 (s, 1H, NH), 8.81 (t, *J* = 5.9 Hz, 1H, NH), 8.21 (s, 1H, NH), 7.70 (s, 1H, triazole), 7.20‐7.15 (m, 6H, H2, H3, H5, H6, H3′, H5′), 7.01 (d, *J* = 8.5 Hz, 2H, H2′, H6′), 5.19 (s, 2H, CH_2_), 5.15‐5.13 (m, 3H, CH_2_, CH), 4.30 (d, *J* = 5.9 Hz, 2H, CH_2_), 4.00 (q, *J* = 7.1 Hz, 2H, CH_2_), 2.29 (s, 3H, CH_3_), 2.27 (s, 3H, CH_3_), 1.12 (t, *J* = 7.1 Hz, 3H, CH_3_) ppm. ^13^CNMR (125 MHz, DMSO‐*d_6_*): 165.9, 165.8, 157.7, 152.7, 148.5, 142.9, 137.9, 136.6, 136.1, 129.4, 127.9, 127.8, 126.6, 114.9, 100.0, 61.5, 59.6, 53.8, 52.1, 42.6, 21.1, 18.3, 14.6 ppm. MS (*m/z*, %): 518 (M^+^, 2), 367 (8), 276 (17), 247 (72), 183 (58), 155 (36), 64 (100). Calcd for C_27_H_30_N_6_O_5_: C, 62.54; H, 5.83; N, 16.21. Found: C, 62.47; H, 5.81; N, 16.48.

### 2.3 MTT Assay

The cell lines used in this study were MCF‐7, human breast adenocarcinoma cell line (NCBI code: C135; ATCC Number: HTB‐22; ECACC Number: 86012803; RRID: CVCL_0031), Hep‐G2, human hepatocellular carcinoma cell line (NCBI code: C158; ATCC: HB‐8065; ECACC: 85011430; RRID: CVCL_0027), SW480, human colon adenocarcinoma cell line (NCBI Code: C506; ATCC: CCL‐228; ECACC: 87092801; RRID: CVCL_0536) and Hek‐293, human embryonic kidney line (NCBI Code: C497; ATCC: CRL‐1573; ECACC: 85120602). All cells were purchased from the Pasteur Institute of Iran. The cytotoxic activity of compounds **8a‐n** was evaluated against human cancer cell lines using the MTT (3‐(4,5‐dimethylthiazol‐2‐yl)‐2,5‐diphenyl tetrazolium bromide) colorimetric assay, as previously described [26, 27]. Absorbance was measured at 570 nm with a reference wavelength of 690 nm. Data were analyzed using GraphPad Prism software, and IC_50_ values were calculated by nonlinear regression analysis.

### 2.4 Cell Cycle Analysis

MCF‐7 cells (1 × 10^5^) were subjected to compound **8n** at the IC_50_ concentration for 72 h. Following treatment, cells were harvested and fixed in 2.5 mL of ice‐cold 70% ethanol for 2 h. Cells were pelleted by centrifugation at 4000 rpm for 2 min, resuspended in 500 μ L PBS containing 0.25% Triton X‐100, and incubated on ice for 15 min. Cells were pelleted again at 4000 rpm for 2 min. Finally, the cells were added to flow cytometry tubes containing 500 μ L PBS, 10 μ g/mL RNase A, and 20 μ g/mL propidium iodide (PI) and incubated for 30 min in the dark. Cell cycle distribution was analyzed using a BD FACSCalibur flow cytometer [28].

### 2.5 Assessment of Apoptosis in MCF‐7 Cells

Annexin V‐FITC/PI detection kit (BD Pharmingen, San Diego, CA, USA) was used to assess apoptosis. MCF‐7 cells were plated in 6‐well plates at a seeding density of 1 × 10^5^ cells/mL and allowed to attach for 24 h before treatment. The test compounds were then added, and cells were exposed for an additional 48 h. Following treatment, cells were detached by trypsinization, collected into 1.5 mL microcentrifuge tubes, and rinsed twice with PBS to remove residual medium. Cell pellets were subsequently labeled with 5 µL Annexin V‐FITC and 5 µL PI, and apoptotic populations were quantified by flow cytometry [24].

### 2.6 Molecular Docking

Docking studies were carried out using the induced‐fit docking (IFD) protocol with the Schrödinger package. Compound **8n** was first converted to a 3D conformation using Maestro. The crystallographic structure of human Eg5 kinesin (PDB: 2IEH) was downloaded from the PDB website and edited through Maestro's Protein Preparation Wizard before use. During this step, the co‐crystallized ligand and water molecules were extracted from the structure, hydrogen atoms were added and their positions optimized, and the protonation states of ionizable residues were assigned according to PROPKA at physiological pH (7.0) [29]. Docking calculations were performed in IFD mode, allowing ligand conformational flexibility, using the OPLS‐2005 force field. The active site was defined within 12 Å of the reference crystallographic ligand. Up to 20 candidate poses were generated per run, using a van der Waals radius of 0.7 Å for the receptor and 0.5 Å for the ligand [30]. Poses exceeding a prime energy threshold of 30 kcal/mol were discarded for further consideration. To confirm the reliability of this docking workflow, a re‐docking validation was carried out. This yielded an RMSD of 0.79 Å between the re‐docked and original crystallographic ligand poses, supporting the accuracy and dependability of the protocol used [31].

### 2.7 Molecular Dynamics Simulation

Following induced‐fit docking, the high‐ranked binding pose of the **8n**‐Eg5 complex was selected for molecular dynamics analysis using Desmond v5.3 within the Schrödinger Maestro platform. The complex was placed at the center of an orthorhombic simulation cell and solvated with explicit SPC water molecules under periodic boundary conditions. The system was neutralized by adding counterions, and physiological ionic strength was achieved with 0.15 M NaCl.

The simulation proceeded through three stages, including energy minimization, equilibration, and production. Initial relaxation of the system was performed using the steepest‐descent algorithm over 2500 minimization steps. During the heating phase, a weak restraining force was imposed on the protein backbone to avoid structural distortion while the temperature was raised from 0 to 300 K. Production phases were then performed within the NPT ensemble, holding atom number, pressure (1.01325 bar), and temperature (300 K) constant, with temperature achieved through a Nosé–Hoover chain thermostat. Electrostatic interactions over long distances were treated using the Particle Mesh Ewald (PME) algorithm and a 9.0 Å cutoff was applied for short‐range Coulombic interactions. All simulations employed the OPLS‐4 force field, with the protein‐ligand complex subjected to a run of 100 ns [32].

### 2.8 Statistical Analysis

One‐way ANOVA with GraphPad Prism 8 software was used for statistical analysis. Data are expressed as the mean ± standard deviation (SD).

## 3 Results and Discussions

### 3.1 Chemistry

The Biginelli reaction was used as an efficient multicomponent strategy to construct the DHPM) core, enabling rapid synthesis of structurally diverse derivatives from readily available starting materials and facilitating subsequent structure–activity relationship (SAR) studies. The synthetic route (Scheme 1) to target compounds **8a‐n** commenced with the preparation of ethyl 6‐methyl‐2‐oxo‐4‐(4‐(prop‐2‐yn‐1‐yloxy)phenyl)‐1,2,3,4‐tetrahydropyrimidine‐5‐carboxylate (**4**). This was achieved through a Biginelli reaction involving urea (**1**), 4‐(prop‐2‐yn‐1‐yloxy)benzaldehyde (**2**) [33], and ethyl acetoacetate (**3**) under reflux conditions in acetic acid (HOAc), with a catalytic amount of HCl.

**SCHEME 1:**
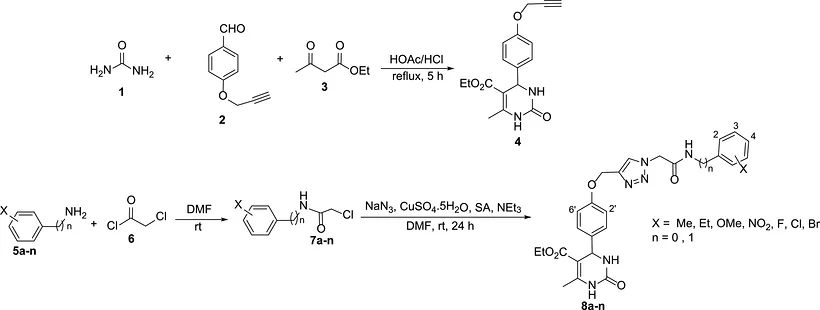
Synthesis of compounds **8a‐n**.

Concurrently, aniline or benzylamine derivatives **5a‐n** were reacted with chloroacetyl chloride (**6**) in DMF at ambient temperature to yield chloroacetamide intermediates **7a‐n** [34]. Subsequently, the target compounds **8a‐n** were synthesized via a Click reaction between compounds **4** and **7a‐n** in DMF. This process utilized sodium azide (NaN_3_), triethylamine (NEt_3_), catalytic amounts of CuSO_4_·5H_2_O, and SA at room temperature for 24 h.

The structure of all synthesized compounds was elucidated by IR and NMR spectroscopy as well as Mass spectrometry, and the purity of the compounds was confirmed by HPLC analysis (Figures S1–S22). For example, the IR spectrum of compound **8n** displayed characteristic absorption bands at 3245 cm^−1^ (NH stretching) and 1725–1695 cm^−1^ (C═O stretching), corroborating the presence of amide and ester functional groups. In the ^1^HNMR spectrum of this compound, the downfield region showed three distinct signals at 9.18, 8.81, and 8.21 ppm, corresponding to the three NH protons, while a singlet at 7.70 ppm was assigned to the 1,2,3‐triazole ring proton. The aromatic protons appeared as a multiplet and a doublet in the region of 7.20–7.01 ppm. The aliphatic region also confirmed an ethyl ester group, evidenced by a quartet at 4.00 ppm and a triplet at 1.12 ppm, along with methylene and methyl singlets integral to the scaffold. The ^13^C NMR spectrum showed 23 distinct resonances, including three carbonyl, 12 aromatic, and 8 aliphatic carbon signals, supporting the proposed structure. The mass spectrum also showed a molecular ion peak at 518.

### 3.2 Structure–Activity Relationship of **8a‐n** as Cytotoxic Agents

The SAR analysis of compounds **8a‐n** evaluated how different substituents influenced cytotoxic activity against cancer cell lines, including HepG2, SW480, and MCF‐7, as well as their selectivity relative to the embryonic kidney noncancerous Hek‐293 cell line (Table 1).

**TABLE 1:** Cytotoxic activity of compounds **8a‐n** ^a^.

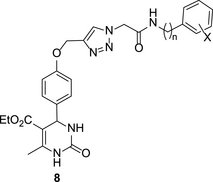						
Compound 8	*X*	*n*	MCF‐7	SW480	Hep G2	Hek‐293
			IC_50_ (µM)	IC_50_ (µM)	IC_50_ (µM)	IC_50_ (µM)
**8a**	H	0	39.10 ± 3.47	83.63 ± 7.67	> 100	> 100
**8b**	4‐Me	0	46.06 ± 6.71	62.26 ± 11.79	> 100	> 100
**8c**	2,3‐diMe	0	44.37 ± 8.05	41.23 ± 5.23	> 100	38.38 ± 2.89
**8d**	2,6‐diMe	0	31.53 ± 0.90	63.26 ± 8.95	> 100	78.26 ± 11.86
**8e**	4‐Et	0	56.33 ± 3.17	69.80 ± 8.49	87.76 ± 6.13	52.98 ± 3.99
**8f**	4‐OMe	0	50.27 ± 5.05	76.47 ± 11.69	> 100	> 100
**8g**	4‐NO_2_	0	40.47 ± 4.01	> 100	> 100	> 100
**8h**	2‐Me‐4‐NO_2_	0	24.71 ± 0.31	73.29 ± 0.87	> 100	> 100
**8i**	2‐F	0	43.26 ± 8.28	> 100	> 100	69.42 ± 7.14
**8j**	4‐Cl	0	67.16 ± 4.09	> 100	> 100	> 100
**8k**	2,4‐diCl	0	> 100	52.73 ± 4.75	> 100	> 100
**8l**	4‐Br	0	> 100	75.64 ± 13.00	> 100	38.51 ± 4.70
**8m**	H	1	34.50 ± 0.92	49.83 ± 1.02	> 100	> 100
**8n**	4‐Me	1	23.70 ± 0.27^b^	57.50 ± 3.49^c^	> 100^c^	50.01 ± 10.20
**Cisplatin**			11.40 ± 3.4	14.85 ± 0.82	24.60 ± 5.1	11.87 ± 0.27

^a^ Data were presented as mean ± SD.

^b^ The comparison between **8n** and cisplatin showed a *p*‐value of 0.0007 in MCF‐7.

^c^ The comparison between **8n** and cisplatin showed a *p*‐value of <0.0001 in SW480 and Hep G2.

The unsubstituted analog, **8a**, showed activity against MCF‐7 cancer cells with an IC_50_ value of 39.10 µM. It displayed lower effectiveness against SW480 cells (IC_50_ = 83.63 µM) and was inactive against HepG2 cells (IC_50_ > 100 µM). To assess the impact of electron‐donating groups, several methyl‐substituted derivatives were synthesized. The 4‐methyl derivative (**8b**) showed moderate activity against MCF‐7 cells (IC_50_ = 46.06 µM). It also showed enhanced activity against SW480 cells (IC_50_ = 62.26 µM) but remained inactive against HepG2 cells. The 2,3‐dimethyl derivative (**8c**) did not improve potency against MCF‐7 cells relative to **8a**, exhibiting an IC_50_ of 44.37 µM compared to 39.10 µM. However, it was more potent than **8b** against SW480 cells. Indeed, in SW480 cells, **8c** (IC_50_ = 41.23 µM) showed a significant improvement over both **8b** and **8a**. Overall, methyl substitutions remained inactive against HepG2 cells (IC_50_ > 100 µM) but selectively enhanced potency in MCF‐7 and SW480 cells, except that 8c reduced selectivity.

Increasing the steric demand of the *para* substituent from methyl to ethyl (**8e**) did not improve activity against MCF‐7 cells. Instead, a modest improvement was observed against HepG2 cells, suggesting the increased alkyl bulk alters the profile in a cell‐line‐dependent manner. Replacing the methyl group with a methoxy substituent (**8f**) did not improve potency either, indicating that adding a hydrogen‐bond acceptor at this position does not contribute favorably and that hydrophobic interactions are more important than increased polarity for this scaffold.

Unique SAR trends were observed for electron‐withdrawing substituents. The *para*‐nitro analog **8g** was active against MCF‐7 but inactive against the other cell lines, indicating that strong electron withdrawal alone is not sufficient for increasing potency. However, the best activity profile in this series was observed with a 2‐Me‐4‐NO_2_ substituent (**8h**), providing improved MCF‐7 potency with little toxicity to Hek‐293 cells. These results indicate that modulating steric and electronic properties is more beneficial than either modification alone, and that the resulting electronic properties may allow more favorable recognition of the biological target.

The effect of halogen substitution also depended strongly on both the type and position of the halogen. Fluorine substitution (**8i**) maintained moderate activity and selectivity, whereas substitution with chlorine (**8j**) or bromine (**8l**) gradually decreased activity on MCF‐7. In particular, the brominated compound, as a larger and more lipophilic analog, was more toxic to Hek‐293 cells. The 2,4‐dichloro derivative (**8k**) shifted the activity toward SW480 cells, indicating that multiple halogen substitutions change the interaction profile and may favor an alternative binding mode.

The antiproliferative activity was enhanced by increasing the linker length from *n* = 0 to *n* = 1. Comparison of the corresponding analog pairs (**8a** vs. **8m** and **8b** vs. **8n)** showed that linker extension resulted  in enhauced activity against both MCF‐7 and SW480 cells, with **8n** being the most potent compound of the series. This improved activity correlates well with the molecular docking data, in which the longer linker provides better orientation of the triazole‐acetamide moiety in the Eg5 allosteric binding pocket to simultaneously interact with key residues such as Glu116, Arg119, Ser120, and Asn122. However, the increased potency of **8n** was accompanied by reduced selectivity for Hek‐293 cells, suggesting that increased conformational flexibility enhances target engagement but also increases nonspecific cytotoxicity.

In conclusion, SAR shows that a balance of steric, electronic, and conformational properties is necessary for optimum biological activity. Among the substitutions studied, **8h** demonstrated the best selectivity profile, while linker extension gave the highest antiproliferative potency (**8n**). These results identify electronic modulation and controlled linker flexibility as the main parameters influencing the anticancer activity of this series.

It is also informative to compare the activity of the most potent derivative, compound **8n**, with previously reported Eg5 inhibitors such as monastrol and ispinesib. Monastrol, a well‐known DHPM‐based Eg5 inhibitor, typically exhibits moderate antiproliferative activity in cancer cell lines with micromolar potency. In contrast, clinically investigated Eg5 inhibitors such as ispinesib display substantially higher potency, often in the nanomolar range. In the present study, compound **8n** demonstrated the strongest cytotoxic activity among the synthesized derivatives, particularly against MCF‐7 cells (IC_50_ = 23.70 ± 0.27 µM). Although its potency is lower than that of highly optimized Eg5 inhibitors such as ispinesib, the activity of **8n** remains comparable to early‐generation DHPM‐based inhibitors like monastrol. Considering its Biginelli‐derived scaffold and synthetic accessibility, compound **8n** may serve as a promising lead structure for further structural optimization toward more potent Eg5 inhibitors with improved anticancer activity.

### 3.3 Mechanism of the Observed SAR Trends

The cytotoxicity across the four cell lines suggests an intracellular target and/or membrane permeation or solvation behavior. The marked potency achieved by extending the linker from *n* = 0 to *n* = 1 (as seen with **8n**) is observed in SW480 and MCF‐7 cell lines, indicating that this conformational freedom is required to overcome steric hindrance and enable the pharmacophore to adopt the proper orientation for the proposed target. A second pattern emerges among the *n* = 0 derivatives, where cell‐line selectivity appears to depend on lipophilicity and electronic properties. Compound **8h** (2‐Me‐4‐NO_2_), which combines moderate lipophilicity with electronic properties, might increase cellular uptake and induce toxicity in MCF‐7 cells. By contrast, the more lipophilic **8l** (4‐Br) showed greater toxicity against the noncancerous Hek‐293 line (IC_50_ = 38.51 µM) while remaining largely inactive against Hep‐G2 and MCF‐7. This may be due to increased lipophilicity favoring normal rather than cancer cell membranes.

However, the strongly polar 4‐OMe substituent in **8f** and 4‐NO_2_ in **8g** may impose a desolvation penalty for membrane crossing, which could explain their diminished potency.

These observations suggest that anticancer efficacy in this series is optimized not by any single structural feature, but by the combined presence of linker flexibility and a polarizability profile.

### 3.4 Cell Cycle Analysis

To elucidate the mechanism underlying the cytotoxicity of compound **8n**, the cell cycle distribution was assessed (Figure 3). Data for untreated control cells revealed that the majority were in the G1 phase (65.82%), while 18.1% were in the S phase and 16.08% were in the G2 phase, indicating a normal cell cycle profile. However, treatment with **8n** dramatically decreased the G1 phase population (32.7%), suggesting that this compound disrupts normal G1 progression. In contrast, we observed a markedly increased S‐phase population (36.4%), indicating that **8n** induces S‐phase arrest by inhibiting DNA replication.

**FIGURE 3:**
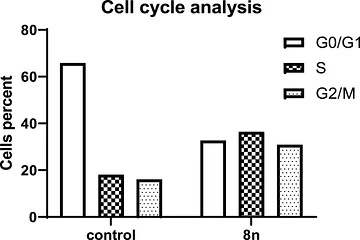
Cell cycle analysis in MCF‐7 cancer cells after 72 h of **8n** treatment.

A large population of cells in the G2/M phase was also observed (30.9%), suggesting an additional block at the G2/M checkpoint that prevents cells from entering mitosis. This indicates that cancer cells cannot properly complete DNA replication or mitosis, potentially leading to cell death.

### 3.5 Apoptosis Analysis

Apoptosis analysis (Figure 4) showed that the viable cell population (Q4) significantly decreased (59.8%) compared with the control (93.0%) after treatment with **8n** at 24 µM.

**FIGURE 4:**
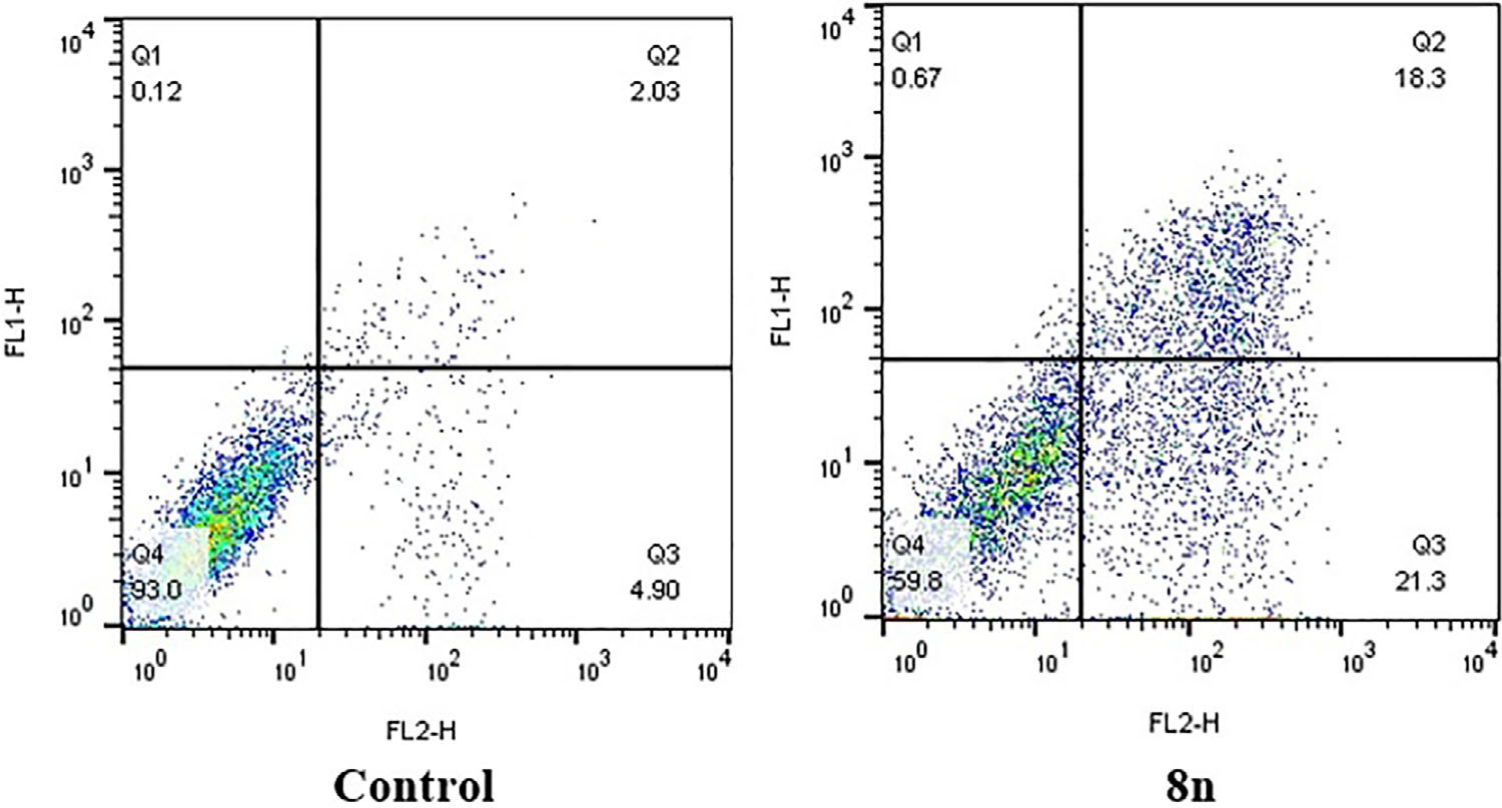
Apoptosis induction in MCF‐7 cancer cells by **8n** versus the control group after 72 h.

The early apoptotic population (Q3) increased from 4.9% to 21.3%, indicating apoptosis induction. Similarly, the late apoptotic population (Q2) increased from 2.0% in the control to 18.3% in the treated cells, further supporting the pro‐apoptotic activity of **8n**. Notably, the necrotic fraction (Q1) remained low (0.67% for **8n** treatment vs. 0.12% for control), confirming that cell death occurred primarily via apoptosis. These observations demonstrate that **8n** induced apoptosis in a regulated manner, markedly increasing both early and late apoptotic populations while reducing cell viability.

### 3.6 Docking Studies

Monastrol and its structural analogs have been extensively studied as mitotic Eg5 inhibitors to elucidate their mechanism of action in the cell cycle.

Eg5, also known as KIF11, is a member of the kinesin‐5 motor protein family and functions as a homotetrameric, slow‐processive motor that moves toward microtubule plus ends. Eg5 plays an essential role in establishing bipolar spindle architecture and ensuring proper chromosome alignment during mitosis.

Given the high structural similarity of **8n** to Monastrol‐based derivatives, this analog was evaluated against Eg5 using molecular docking and in silico simulations. Initially, the crystallographic ligand with a monastrol‐based structure was docked into the Eg5 active site to validate the docking protocol and identify key interacting residues. The molecular docking results are illustrated in Figure 5. The NH group of the tetrahydropyrimidine moiety showed a hydrogen‐bonding interaction with Gly116, while the hydroxyl of the 3‐hydroxyphenyl formed an additional hydrogen‐bonding interaction with Leu214.

**FIGURE 5:**
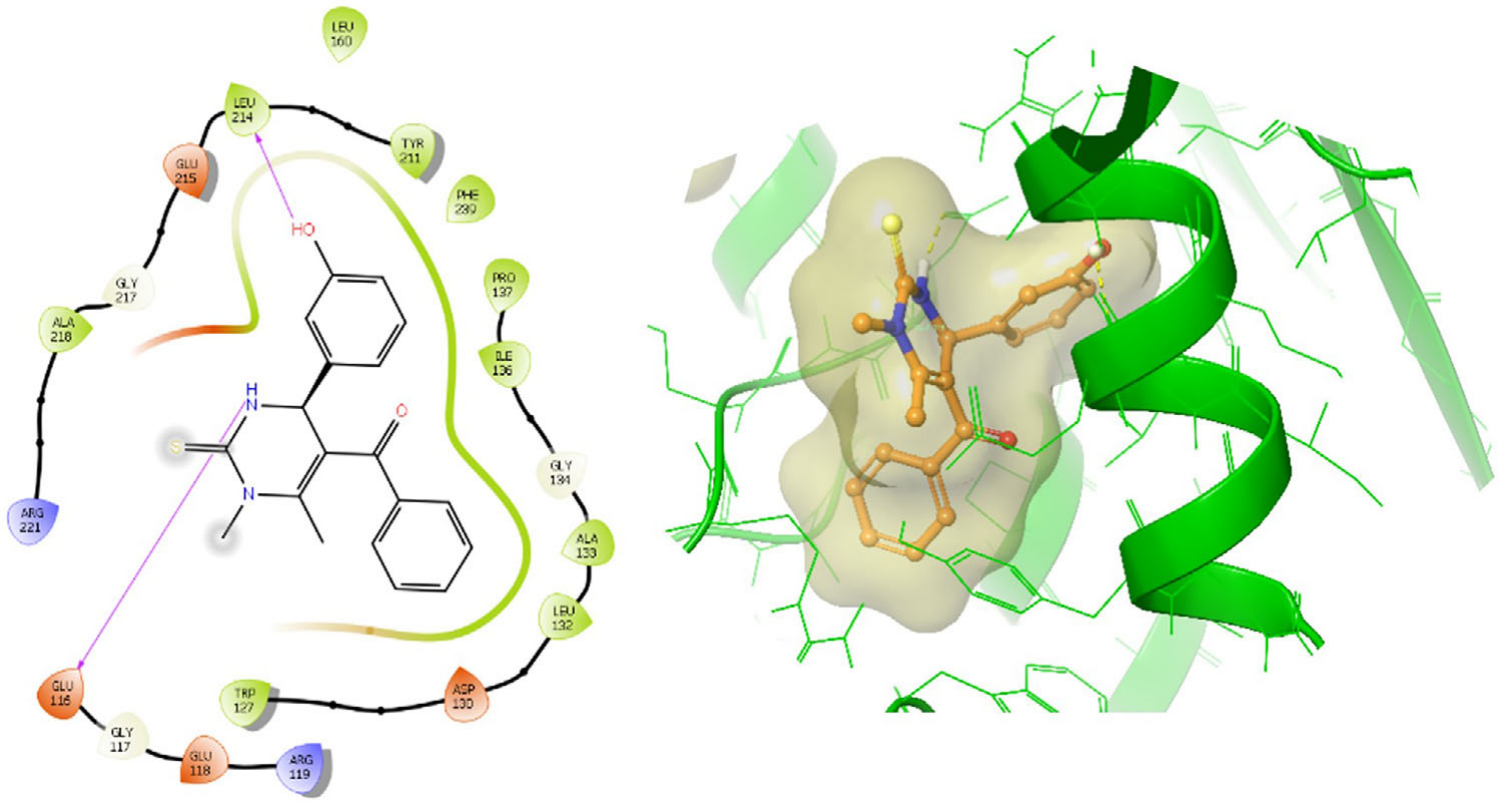
2D and 3D interactions of the monastrol‐based inhibitor in the Eg5 active site.

Subsequent molecular docking studies were performed on the most potent analogs. In compound **8n**, the NH group of the tetrahydropyrimidine showed hydrogen‐bonding interaction with Ser120, while the carbonyl group formed two hydrogen‐bonding interactions with Asn122 and Glu123. Additionally, the amide moiety attached to the 4‐methylphenyl group formed an additional hydrogen‐bonding interaction with Glu116 (Figure 6).

**FIGURE 6:**
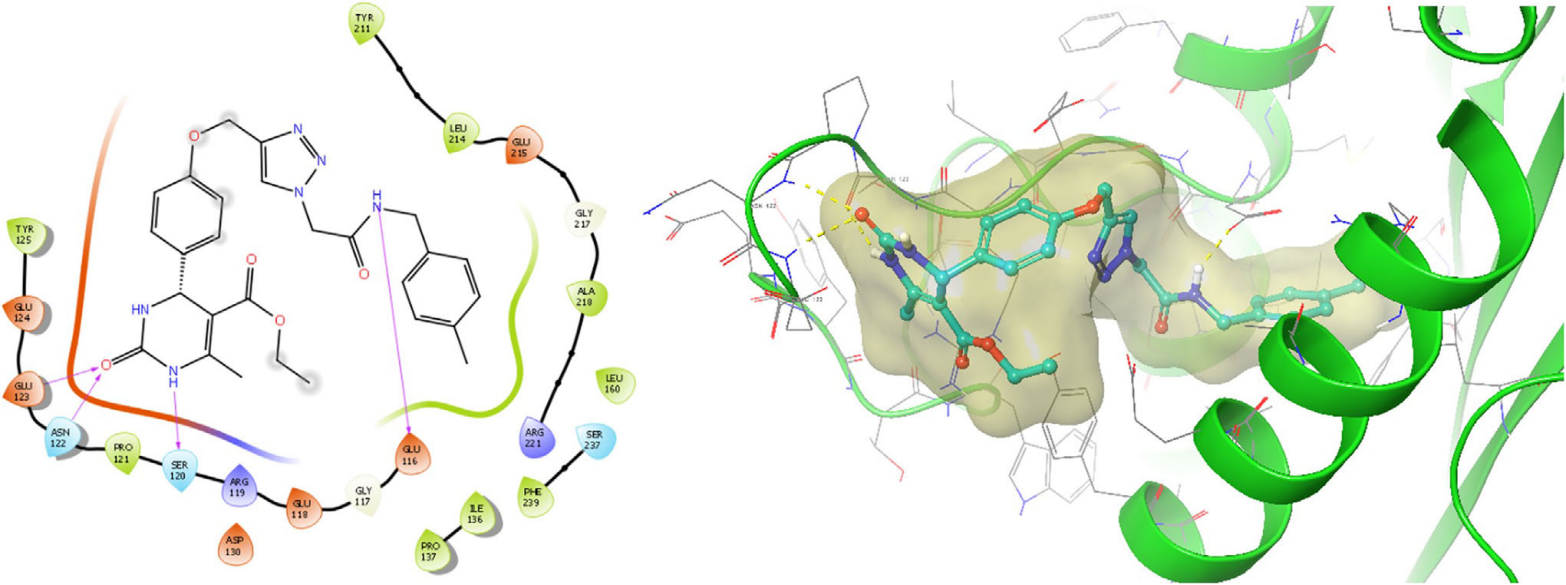
**2**D and 3D interactions of **8n** in the Eg5 active site.

### 3.7 Molecular Dynamics

Molecular dynamics simulations assessed the stability of the **8n**‐Eg5 complex over time. The RMSD (root mean square deviation) plot demonstrates the structural stability of Eg5 in both its apo form and in complex with **8n** (Lig‐Eg5) over a 100 ns MD simulation. Apo‐Eg5 showed a higher RMSD of 3.5 Å. In contrast, the **8n**‐Eg5 complex showed a lower RMSD of approximately 2.5 Å, suggesting that **8n** contributes to Eg5 structural stability. This stabilization effect implies a potential role for **8n** in maintaining the conformational integrity of Eg5 (Figure 7).

**FIGURE 7:**
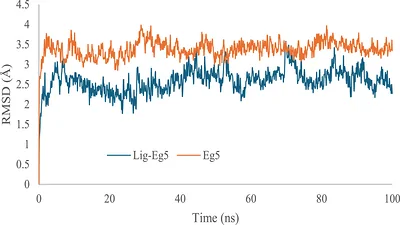
RMSD of Eg5 versus **8n**‐Eg5.

The root mean square fluctuation (RMSF) plot illustrates the flexibility of individual Eg5 residues in the presence and absence of **8n**. The apo form of Eg5 (orange line) shows higher fluctuations in several regions, including 36‐54, 100‐116, and 160‐180, than the **8n**‐Eg5 complex (blue line). These regions correspond to active site regions. The reduced flexibility in these residues showed that ligand binding stabilizes the protein structure, likely by reducing the conformational flexibility in these regions. This stabilization was further validated by analyzing interactions persisting for more than 30% of the simulation time.

Analysis of the MD simulation of compound **8n** within the Eg5 active site highlighted several key stabilizing interactions (Figure 8a). The NH group of the tetrahydropyrimidine ring formed an H‐bond with Asn122 (Figure 8b), while its carbonyl group (C═O) formed a direct H‐bond with Glu123 and a water‐mediated H‐bond with Ser120. Moreover, the ethyl acetoacetate moiety formed an H‐bond with Glu215 mediated by a water molecule. Notably, Arg119 interacted with the methoxy linker and formed two hydrogen bonds with the carbonyl oxygen of the triazole acetamide group. Additionally, a π‐cation interaction between Arg119 and the triazole further stabilized the ligand within the active pocket. Furthermore, the NH group of the acetamide moiety formed two H‐bonds with Glu116. Collectively, these interactions contribute significantly to the binding affinity and stability of **8n**, reinforcing its potential as a potent Eg5 inhibitor.

**FIGURE 8:**
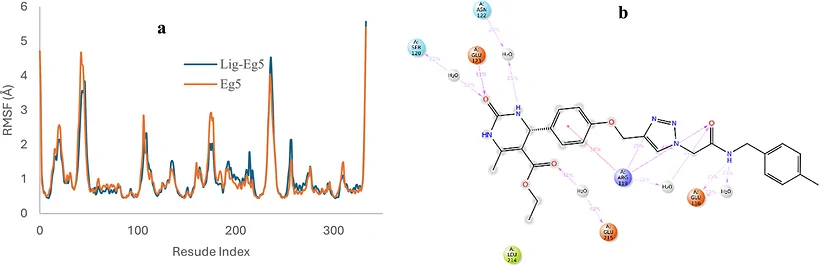
(a) RMSF of Eg5 versus **8n**‐Eg5; (**b**) 2D interactions of **8n**‐Eg5 recorded for more than 30% of the simulation time.

These computational results are not biochemical or cellular validation of Eg5 as the molecular target, but rather a prediction.

## 4 Conclusion

In this study, a novel series of Biginelli‐based 1,2,3‐triazole hybrids, **8a‐n**, was designed, synthesized, and evaluated for their anticancer potential. Among the synthesized compounds, **8n** showed the highest cytotoxic activity against the MCF‐7 breast cancer cell line (IC_50_ = 23.70 µM), moderate activity against SW480 cells (IC_50_ = 57.5 µM), and no significant activity against HepG2 cells. Cell cycle analysis of MCF‐7 cells treated with **8n** revealed arrest in the S and G2/M phases. Furthermore, **8n** significantly induced apoptosis, as determined by Annexin V‐FITC/PI staining. To further elucidate its mechanism of action, molecular docking and MD simulations were performed, identifying Eg5 as a plausible molecular target. Compound **8n** bound the Eg5 active site through key hydrogen bonds and hydrophobic interactions, and MD simulations confirmed the stability of the **8n**‐Eg5 complex. Together, these results highlight compound **8n** as a promising anticancer candidate through mechanisms of cell cycle arrest and apoptosis induction, warranting further preclinical evaluation.

A limitation of this study is that we did not experimentally validate Eg5 inhibition through biochemical or cell‐based target‐engagement assays. Further studies with such experiments are justified to confirm that Eg5 is the key molecular target of these compounds.

## Author Contributions

**Aida Iraji** supervised the biological assays and performed the in silico studies. **Sara Moghadam Farid** synthesized the compounds. **Mohammadreza Eftekharian** conducted the biological assays. **Paniz Naseri** and **Mohammad Mahdavi** contributed to compound synthesis. **Mina Saeedi** and **Tahmineh Akbarzadeh** designed the project and supervised all stages of the study.

## Conflicts of Interest

The authors declare no conflicts of interest.

## Supporting information




**Supporting File 1**: cbdv71810‐sup‐0001‐SuppMat.docx.

## Data Availability

Data will be made available from the corresponding authors upon reasonable request.
