# Supplementary material for: Design and Synthesis of Novel Biginelli Adducts: Anticancer Activity and In Silico Study
Source: Chem Biodivers. 2026 Oct 12;23(10):e71810. doi: 10.1002/cbdv.71810 (PMC13655646; doi:10.1002/cbdv.71810)
Supplement: Supplementary file 1 [file CBDV-23-e71810-s001.docx]

**Supplementary file**

**Design and Synthesis of Novel Biginelli Adducts: Anticancer Activity and *In Silico* Study**

**Copies of NMR spectra of synthesized compounds ……………………………………...S2-S15**

**HPLC Chromatograms ……………………………………………………….....………S16-S20**

**Figure S1. ^1^H- and ^13^C- NMR spectra of compound 8a**

**Figure S2. ^1^H- and ^13^C- NMR spectra of compound 8b**

**Figure S3. ^1^H- and ^13^C- NMR spectra of compound 8c**

**Figure S4. ^1^H- and ^13^C- NMR spectra of compound 8d**

**Figure S5. ^1^H- and ^13^C- NMR spectra of compound 8e**

**Figure S6. ^1^H- and ^13^C- NMR spectra of compound 8f**

**Figure S7. ^1^H- and ^13^C- NMR spectra of compound 8g**

**Figure S8. ^1^H- and ^13^C- NMR spectra of compound 8h**

**Figure S9. ^1^H- and ^13^C- NMR spectra of compound 8i**

**Figure S10. ^1^H- and ^13^C- NMR spectra of compound 8j**

**Figure S11. ^1^H- and ^13^C- NMR spectra of compound 8k**

**Figure S12. ^1^H- and ^13^C- NMR spectra of compound 8l**

**Figure S13. ^1^H- and ^13^C- NMR spectra of compound 8m**

**Figure S14. ^1^H- and ^13^C- NMR spectra of compound 8n**

**HPLC chromatography**

**Figure S15. HPLC chromatography: Blank**

**Figure S16. HPLC chromatography of compound 8c**

**Figure S17. HPLC chromatography of compound 8d**

**Figure S18. HPLC chromatography of compound 8g**

**Figure S19. HPLC chromatography of compound 8h**

**Figure S20. HPLC chromatography of compound 8i**

**Figure S21. HPLC chromatography of compound 8m**

**Figure S22. HPLC chromatography of compound 8n**
